# Erosive tooth wear inhibition by hybrid coatings with encapsulated fluoride and stannous ions

**DOI:** 10.1007/s10856-021-06554-2

**Published:** 2021-07-01

**Authors:** Sávio José Cardoso Bezerra, Ítallo Emídio Lira Viana, Idalina Vieira Aoki, Maria Angela Pita Sobral, Alessandra Buhler Borges, Anderson T. Hara, Taís Scaramucci

**Affiliations:** 1 Department of Restorative Dentistry, University of São Paulo, School of Dentistry, São Paulo, São Paulo Brazil; 2 Department of Chemical Engineering, Polytechnic School, São Paulo University—USP, São Paulo, São Paulo Brazil; 3 Department of Restorative Dentistry, Institute of Science and Technology, São Paulo State University—UNESP, São José dos Campos, São Paulo Brazil; 4 Department of Cariology, Operative Dentistry and Dental Public Health, Indiana University School of Dentistry, Indianapolis, IN USA

## Abstract

This study aimed to formulate a hybrid coating material (HC) and to modify this HC with fluoride (NaF) and stannous (SnCl_2_) ions, directly or encapsulated in nano containers, testing the effects of these materials against dental erosion and erosion–abrasion. Enamel and dentin specimens were treated with the HCs, and then tested in erosion or erosion–abrasion cycling models of 5 days (*n* = 10 for each substrate, for each model). Deionized water was the negative control, and a fluoride varnish, the positive control. Surface loss (SL, in µm) was evaluated with an optical profilometer, and data were statistically analyzed (*α* = 0.05). For enamel, in erosion, the positive control and HC without additives showed significantly lower SL than the negative control (*p* = 0.003 and *p* = 0.001). In erosion–abrasion, none of the groups differed from the negative control (*p* > 0.05). For dentin, in erosion, the positive control, HC without additives, HC with non-encapsulated F, and HC with encapsulated F + Sn showed lower SL than the negative control (*p* < 0.05). In erosion–abrasion, none of the groups differed significantly from the negative control (*p* < 0.05). HC without additives showed a promising potential for protecting the teeth against dental erosion (with upward trend for improved protection on dentin), but not against erosion–abrasion. The presence of additives did not improve the protective effect of the HC, on both substrates.

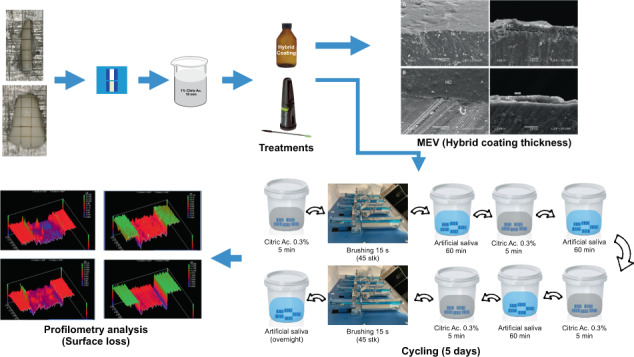

## Introduction

Erosive tooth wear is the gradual loss of dental hard tissues through a multifactorial process that involves the interplay between chemical, biological, and behavioral factors [1]. It is a common condition with global prevalence estimated between 30 and 50% in deciduous teeth, and between 20 and 45% in permanent teeth [2]. Considering the multifactorial nature of this condition, its management should consider the risk factors involved [3], and the level of severity of the existing lesions. The first preventive action recommends reducing or eliminating exposure of the teeth to erosive acids of both extrinsic and intrinsic origin [3, 4]. The topical application of fluoridated products has also been suggested [5].

Sodium fluoride has shown some efficacy against dental erosion, which is most likely achieved by physically protecting the tooth surfaces with deposits [5]. More accentuated protection has been observed with stannous fluoride or the combination between sodium fluoride and stannous chloride [5–7]. In addition to forming surface deposits [8], under the dynamic conditions of demineralization and mineral deposition, stannous can penetrate a few micrometers into tooth surfaces, increasing their acid resistance [9, 10]. Nevertheless, even when using products containing the combination between sodium fluoride and stannous chloride, frequent application is desirable, considering the constant exposure of teeth to erosive challenges [5]. In this regard, a material that could increase the availability of these ions in the oral environment and create an extra mechanical barrier against the erosive acids would be of great value for controlling erosive tooth wear.

In surface engineering, a thin, transparent, and environmentally friendly coating is usually applied over the surface of metals to avoid corrosion, and to help paints to adhere to these surfaces [11]. These coatings can be formulated with inorganic compounds only or with both inorganic and organic compounds (hybrid). They have anti-staining and optical properties, thermal stability, chemical and biological resistance [12]. The incorporation of organic compounds allows the formation of more flexible and functional coatings that have increased compatibility with different organic materials [13]. In dentistry, the use of hybrid coatings has not been fully explored, despite their large number of promising applications, such as preventing staining of resin-based dentures [12, 14], and use as antibacterial agents [14].

It has been hypothesized that the hybrid coatings would be capable of adhering to tooth surfaces after an alkaline treatment [15], since ions OH^−^ are created on tooth surfaces at the calcium sites. The hybrid coatings are derived from hybrid solutions that contain silanol, which retains the Si–OH groups capable of interacting with the Ca–OH groups of tooth surfaces by means of low energy bonding, as hydrogen bonds. After the cure, crosslinking occurs in the hybrid solution resulting in chemically stable siloxane –Si–O–Si and –Ca–O–Si– bonds with the tooth surfaces. This would allow the hybrid coatings to act as mechanical barriers against the acid challenges, with the advantages of the coating being a thin and transparent film that would be easily applicable and have a low production cost. Although the hybrid coatings would be expected to remain bonded to the tooth surfaces for a long period, defects can occur, especially considering the constant chemical and mechanical challenges existent in the oral cavity environment [16]. In this study, we theorized that the incorporation of fluoride and stannous ions, known anti-erosion agents [17–19], into the hybrid coating could counteract dental erosion and erosion–abrasion specifically in the HC defects, prolonging its overall protective effect. We also suggested that the addition of the anti-erosive agents in nano-capsules could increase their availability, allowing for a controlled and sustained release [20]. In addition, nano-capsules would provide an increase in the mechanical resistance of the HC [21].

This study aimed to formulate a hybrid coating and to test its effects against enamel and dentin dental erosion and erosion–abrasion. It also aimed to investigate the addition of anti-erosive agents, directly or encapsulated in halloysite and bentonite clay, to the hybrid coating and to test the effects of these modified hybrid coatings against dental erosion and erosion–abrasion. The hypotheses of this study were: (1) the hybrid coating would act as a mechanical barrier, protecting the enamel and dentin against dental erosion and erosion–abrasion and (2) the hybrid coatings modified with fluoride and stannous would show improved protection against dental erosion and erosion–abrasion when compared with the non-modified hybrid coating.

## Materials and methods

### Study design

This study followed a 7 × 2 factorial design, considering: (1) surface treatments (at 7 levels): deionized water—negative control; commercial fluoride varnish—positive control; hybrid coating—HC; HC with fluoride; HC with fluoride plus stannous ions; HC with encapsulated fluoride; and HC with encapsulated fluoride and stannous; and (2) type of cycling models (at 2 levels): erosion and erosion–abrasion, as experimental factors. These treatments were tested in a dental erosion or erosion–abrasion model, using previously eroded bovine enamel (*n* = 10 for each model) and dentin (*n* = 10 for each model) specimens. The response variable was tooth surface loss (SL, in μm), measured at the end of the cycling protocol. All procedures were performed in random and blind sequences.

### Specimen preparation

Enamel and dentin slabs (4 × 4 × 2 mm) were obtained from bovine incisors, using a microtome (Isomet, Buehler, Lake Bluff, IL, USA). The specimens were embedded in acrylic resin (Varidur, Buehler), and the resulting blocks were ground flat and polished with Al_2_O_3_ papers grit #500, 1200, 2400, and 4000 (MD-Fuga, Struers Inc, Cleveland, OH, USA), under water cooling, and polishing cloth and diamond suspension (1 μm; Struers Inc.). At the end of the polishing procedures, the specimens were sonicated in distilled water for 3 min. The specimens with cracks or any other surface defects were discarded. Unplasticized polyvinyl chloride tapes were then placed on the polished surfaces of the selected specimens, leaving a central window of 4 × 1 mm exposed.

### Initial dental erosion lesion

To simulate the condition of a subject that already has signs of erosive tooth wear, an initial erosion lesion was created by immersing the specimens in 1% citric acid solution (pH of ~2.4), at room temperature, for 10 min [22]. After this, specimens were rinsed with deionized water and the tapes were removed. The specimens were then submitted to profilometric analysis with an optical profilometer (Proscan 2100, Scantron, Venture Way, Tauton, UK). An area of 2-mm long in the *x*-axis and 1-mm wide in the *y*-axis was scanned, using the S11/03 sensor, with vertical resolution of 0.012 µm. In the *x*-axis, the step size was 0.01 mm and the number of steps of 200. In the *y*-axis, the step size was 0.1 and the number of steps 10. The depth of the lesion area was calculated, based on subtracting the mean height of the test area from the mean height of the two reference areas, using a specific software (Proscan Application software v. 2.0.17), in order to select those with a lesion size between 2 and 5 µm for the two substrates. The mean (SD) of the SL after creating the initial lesion was 3.00 (0.64) for enamel and 4.03 µm (0.62) for dentin.

### Experimental hybrid coatings

The hybrid solution was prepared as previously described [15, 23], and contained a gamma-aminopropyltriethoxysilane (γ-APS), 3-glycidoxypropyltrimethoxysilane (GPTMS), and tetra ethylorthosilicate (TEOS). In this hybrid solution, functional alkoxides (γ-APS and GPTMS) modified the inorganic precursor (TEOS), forming a structure with organic–inorganic properties. GPTMS was the monomer and coupling agent and γ-APS, the crosslinking promoter [23]. Briefly, TEOS and GPTMS were incorporated into an alcohol plus water solution and kept for 72 h under agitation to promote the hydrolysis of the components. Afterwards, γ-APS was incorporated into the solution. The resulting solution was diluted with deionized water in the ratio of 1:3. For the hybrid coating with fluoride, NaF (Sigma-Aldrich Co., St. Louis, MO, USA) was added to the hybrid solution in a concentration of 0.1 g per 10 ml, resulting in 4520 ppm of F^−^. For the hybrid coating with fluoride and stannous, NaF was added in same concentration and SnCl_2_ (Sigma-Aldrich Co., St. Louis, MO, USA) in the concentration of 0.1 g per 10 ml, resulting in the concentration of 6261 ppm of Sn^2+^. To this hybrid solution, gluconic acid in the concentration of 0.28 per 10 ml was also added for stability purposes. These were the maximum concentrations that it was possible to add. The description of fluoride and stannous encapsulation follows below. For the hybrid coating with encapsulated fluoride, 0.05 g of halloysite loaded with fluoride was added per ml of the hybrid solution, resulting in a concentration of 50,000 ppm of halloysite. For the hybrid solution with encapsulated fluoride and stannous, 0.05 g of both loaded clays (halloysite with fluorides and bentonite with stannous ions) were added per ml of the hybrid solution.

### Encapsulation and release of F^−^ and Sn^2+^

To encapsulate F^−^, a NaF solution of 15,000 ppm F^−^ was prepared. The fluoride was encapsulated in halloysite nano tubes, by adding 12 g of halloysite to 100 ml of the NaF solution. The resulting suspension was kept under vacuum for 30 min and then centrifuged (3000 rpm for 5 min). The supernatant was removed and stored. The resulting mass was washed with deionized water and centrifuged again. This procedure was repeated another three times, once a day. Afterwards, the halloysite was left to dry at 45 °C for 24 h.

To encapsulate the Sn^2+^, a SnCl_2_ solution containing 10,000 ppm Sn^2+^ was used, and 12 g of bentonite was added to 100 ml of this SnCl_2_ solution. The resulting suspension was kept under agitation for 24 h, then centrifuged (3000 rpm for 5 min). The supernatant was removed, and the mass was washed with deionized water and centrifuged again. This daily procedure was repeated for 3 additional days. Afterwards, the bentonite was left to dry at 45 °C for 24 h.

To confirm that the encapsulation process was correct and the nano-capsules were able to release the agents into different media, 1 g of each of the clays was added on 100 ml of neutral solution (artificial saliva) or acid solution (citric acid 0.3%, pH = 2.6) under continuous agitation. Aliquots of 1 ml of each solution were collected after 1, 3, 5, 12, 24, and 48 h. The fluoride release (in µg/g of halloysite) was tested with selective fluoride ion electrode, added the same amount of TISAB II for each aliquot, in triplicate. The Sn^2+^ release (in mg/g of bentonite) was analyzed by Optical Emission Spectrometry, in triplicates. The data were processed with specific software ICP Expert II Software (Agilent Technologies Inc., Wilmington, USA).

### Application of the treatments

After the assessment of the initial lesion, the tapes were repositioned and the specimens were randomly allocated into the experimental groups (*n* = 10 for each substrate, in each of the cycling models), as described in Table 1. The commercial fluoride varnish (Fluor Protector) was applied in accordance with the manufacturer’s instructions. The hybrid coatings were applied according to a protocol established in pilot studies (not shown). First, the alkaline treatment was performed by exposing the specimens to a 0.05-M NaOH solution (pH of ~12.9) for 10 min, followed by rinsing with deionized water and drying. The hybrid solutions were applied with a disposable applicator, in two layers; after application of the first layer, the solution was allowed to dry for 4 min, and after the second application, 4 min was again allowed to elapse before curing each layer, which was performed with the heat from a light curing device (Valo, Ultradent, South Jordan, UT, USA) for 60 s, at irradiance of 1000 mW/cm^2^. To verify the presence of the hybrid coating on the tooth surfaces, three additional enamel and dentin specimens were prepared and analyzed by scanning electron microscopy (JEOL 6460LV, Akishima, Tokyo, Japan). The specimens were treated with the hybrid coating without additives, as previously described, and were sectioned with a diamond disk to obtain a cross-sectional view of the hybrid coat. The surfaces were gold-sputtered, and the scans were performed at 500× and 2000× magnification. The film thickness was measured in three different randomly chosen regions.

**Table 1:** Experimental groups, codes, and its respective surface treatments

Groups	Codes	Description
Negative control	C−	Deionized water application
Positive control	C+	Fluor Protector, 1000 ppm F^−^ (Ivoclair Vivadent Ltda. Zurique, Switzerland)
Hybrid coating	HC	TEOS/GPTMS/Y-APS (TEOS tetraethylsilane, GPTMS 3-glicidoxypropyltrimethoxy silane, γ-APS gamma-aminopropyltrimethoxysilane) solution
Hybrid coating modified by fluoride	HC + F	NaF was added to the hybrid coating solution in the concentration of 0.1 g per 10 ml, resulting in 4520 ppm F^−^
Hybrid coating modified by fluoride and stannous	HC + F + Sn	NaF was added to the hybrid coating solution in the concentration of 0.1 g per 10 ml, resulting in 4520 ppm F^−^, and SnCl_2_ in the concentration of 0.1 g per 10 ml, resulting in the concentration of 6261 ppm de Sn^2+^
Hybrid coating modified by encapsulated fluoride	H + eF	0.5 g of the halloysite loaded with F^−^ was added to the 10 ml of the hybrid solution.
Hybrid coating modified by encapsulated fluoride and stannous	H + eF + eSn	0.5 g of the halloysite loaded with F^−^ and 0.5 g of bentonite loaded with Sn^2+^ were added to the 10 ml of the hybrid solution

### Erosive and erosive–abrasive cycling

The dental erosion cycling was carried out according to a previously described protocol [24]. The specimens were immersed in 4 ml of 0.3% citric acid solution (natural pH of ~2.6) for 5 min, without agitation, followed by 60-min exposure to artificial saliva under gentle agitation at 150 rpm [25]. This procedure was repeated four times a day, for 5 days. All the procedures were carried out at room temperature. The acid was renewed after each exposure. The artificial saliva was changed once a day, at the beginning of the cycling procedure. The tooth erosion–abrasion cycling was carried out according to the same protocol, but the specimens were brushed after the first and last erosive challenges, using a slurry of dentifrice (Crest Cavity Protection—Procter & Gamble, Cincinnati, OH, USA) and deionized water, in a ratio of 1:3. Brushing was performed for 15 s (45 strokes), using a toothbrushing machine and toothbrushes with soft bristles (Oral-B, Procter & Gamble, Cincinnati, OH, USA). Total time of exposure to the slurries was 2 min. After brushing, the specimens were rinsed with deionized water. The specimens of the tooth erosion model were only exposed to the dentifrice slurries, but not brushed. In the overnight period, the specimens were stored in artificial saliva, under gentle agitation.

### SL assessment

On conclusion of the cycling protocols, the tapes were removed from the specimen surfaces, which were analyzed for SL, by using an optical profilometer, as described in “Initial dental erosion lesion”.

### Statistical analysis

SL data for enamel and dentin were analyzed independently. Data were tested for normal distribution and homoscedasticity by means of the Shapiro–Wilk and Brown Forsythe tests, respectively. Considering that data did not follow a normal distribution, they were evaluated by using the Kruskal–Wallis, Dunn and Mann–Whitney tests, considering a level of significance of 5%. The data of ions release were analyzed independently. Considering that the data followed a normal distribution and were homogenous, they were evaluated by the Student *t* test, considering a level of significance of 5%. The SigmaPlot 13 software (Systat Software Inc., San Jose, CA, USA) was used for the calculations.

## Results

**Fig. 1:**
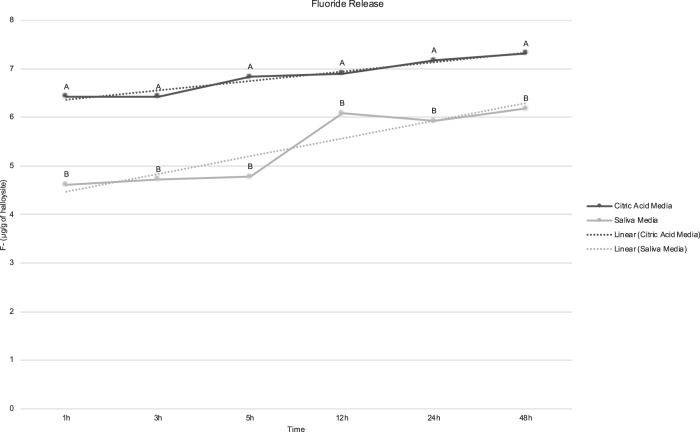
Mean of fluoride released by halloysite (in µg/g of halloysite) on both analyzed media. Different letters denote significant difference between media, for each time (*p* < 0.001)

**Fig. 2:**
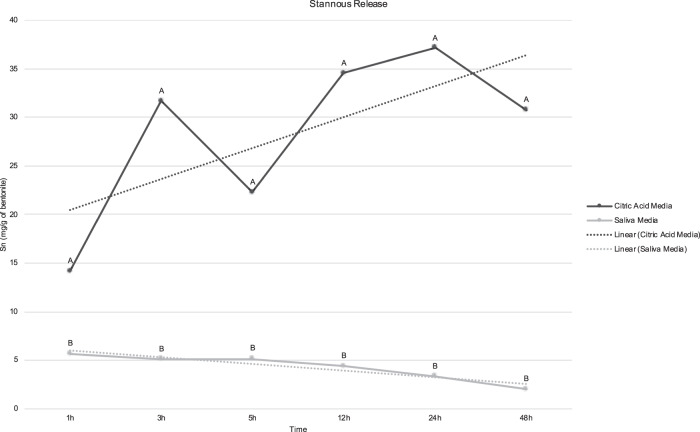
Mean of stannous released by bentonite (in mg/g of bentonite) on both analyzed media. Different letters denote significant difference between media, for each time (*p* < 0.001)

The release analysis confirm that the nano-capsules were able to release the agent on both media (Figs. 1 and 2). The release was greater on acid media than neutral, for both agents.

Table 2 shows the medians (IQ) of enamel SL for the two cycling models. In the erosive model, Fluor Protector and Hybrid Coating were observed to be the only groups that showed lower SL values than the negative control (*p* = 0.003 and *p* = 0.001, respectively), without significant differences between them (*p* = 1). Fluor protector did not differ significantly from Groups HC + F + Sn and HC + eF (*p* = 0.061 and *p* = 0.051, respectively). There were no significant differences among the other groups, and they also did not differ significantly from Groups HC + F + Sn and HC + eF (*p* > 0.05). In the erosive–abrasive model, Fluor Protector showed significantly lower SL values than the other groups (*p* < 0.05), but it did not differ from negative control (*p* = 0.055). SL values of the other groups did not differ significantly among them and in comparison with the negative control (*p* > 0.05). When comparing the cycling models, for each group, Fluor Protector was the only group that showed no significant difference between the two models (*p* = 0.121). The other groups had higher SL values in the tooth erosion–abrasion model in comparison with the tooth erosion (*p* < 0.001).

**Table 2:** Medians and interquartile intervals of enamel surface loss (in μm) at the erosion and erosion–abrasion cycling (*n* = 10, for each cycling model)

Groups	Erosion–abrasion			Erosion		
	Median	IQ		Median	IQ	
C+	−2.75	(−5.27/4.30)	Aa	0.81	(−0.96/2.95)	ABa
HC	−9.02	(−9.96/−7.90)	Bb	0.87	(−0.46/3.27)	Aa
HC + F	−10.21	(−10.64/−9.49)	Bb	−2.66	(−4.52/−1.55)	Ca
HC + eF	−9.64	(−10.54/−8.47)	Bb	−3.13	(−4.52/−1.87)	BCa
HC + eF + eSn	−10.03	(−11.44/−8.61)	Bb	−3.92	(−4.45/−2.63)	Ca
C−	−8.81	(−9.95/−8.53)	ABb	−3.45	(−3.71/−2.96)	Ca
HC + F + Sn	−9.98	(−10.62/−8.32)	Bb	−2.84	(−3.68/−1.52)	BCa

In columns, different uppercase letters denote significant difference among groups, within model (*p* < 0.05). In rows, different lowercase letters denote significant difference between models, within groups (*p* < 0.05).

Table 3 shows the medians (IQ) of dentin SL for the two cycling models. For the erosive model, groups Hybrid Coating, HC + eF+eSn, Fluor Protector, and HC + F did not differ significantly (*p* > 0.05), but they showed significantly lower SL values than the negative control (*p* < 0.05). Groups HC + eF and HC + F + Sn did not differ significantly from the negative control (*p* = 0.107 and *p* = 1, respectively). In the erosive–abrasive model, none of the groups showed significantly lower SL values than negative control (*p* > 0.05), but Hybrid Coating and Fluor Protector had significantly lower SL values than HC + F + Sn (*p* = 0.009 and *p* = 0.021, respectively). Only Groups Fluor Protector and negative control showed no significant difference between the two models (*p* = 0.162 e *p* = 0.970). For the other groups, dental erosion–abrasion promoted significantly higher SL values than erosion (*p* < 0.01).

**Table 3:** Medians and interquartile intervals of dentin surface loss (in μm) at the erosion and erosion–abrasion cycling (*n* = 10, for each model)

Groups	Erosion–abrasion			Erosion		
	Median	IQ		Median	IQ	
C+	−2.95	(−5.07/−0.62)	Aa	−1.37	(−3.01/0.67)	ABa
HC	−4.05	(−4.26/−3.76)	Ab	−0.05	(−2.01/1.49)	Aa
HC + F	−4.10	(−5.24/−2.75)	ABb	−2.36	(−3.28/−1.65)	ABa
HC + eF	−4.35	(−5.48/−4.07)	ABb	−3.23	(−4.15/−1.49)	ABCa
HC + eF + eSn	−5.17	(−5.73/−4.22)	ABb	−1.19	(−4.09/−0.23)	Aa
C−	−5.14	(−5.88/−4.56)	ABa	−5.41	(−5.70/−4.38)	Ca
HC + F + Sn	−6.40	(−6.86/−5.61)	Bb	−4.13	(−4.79/−3.47)	BCa

In columns, different uppercase letters denote significant difference among groups, within model (*p* < 0.05). In rows, different lowercase letters denote significant difference between models, within groups (*p* < 0.05)

**Fig. 3:**
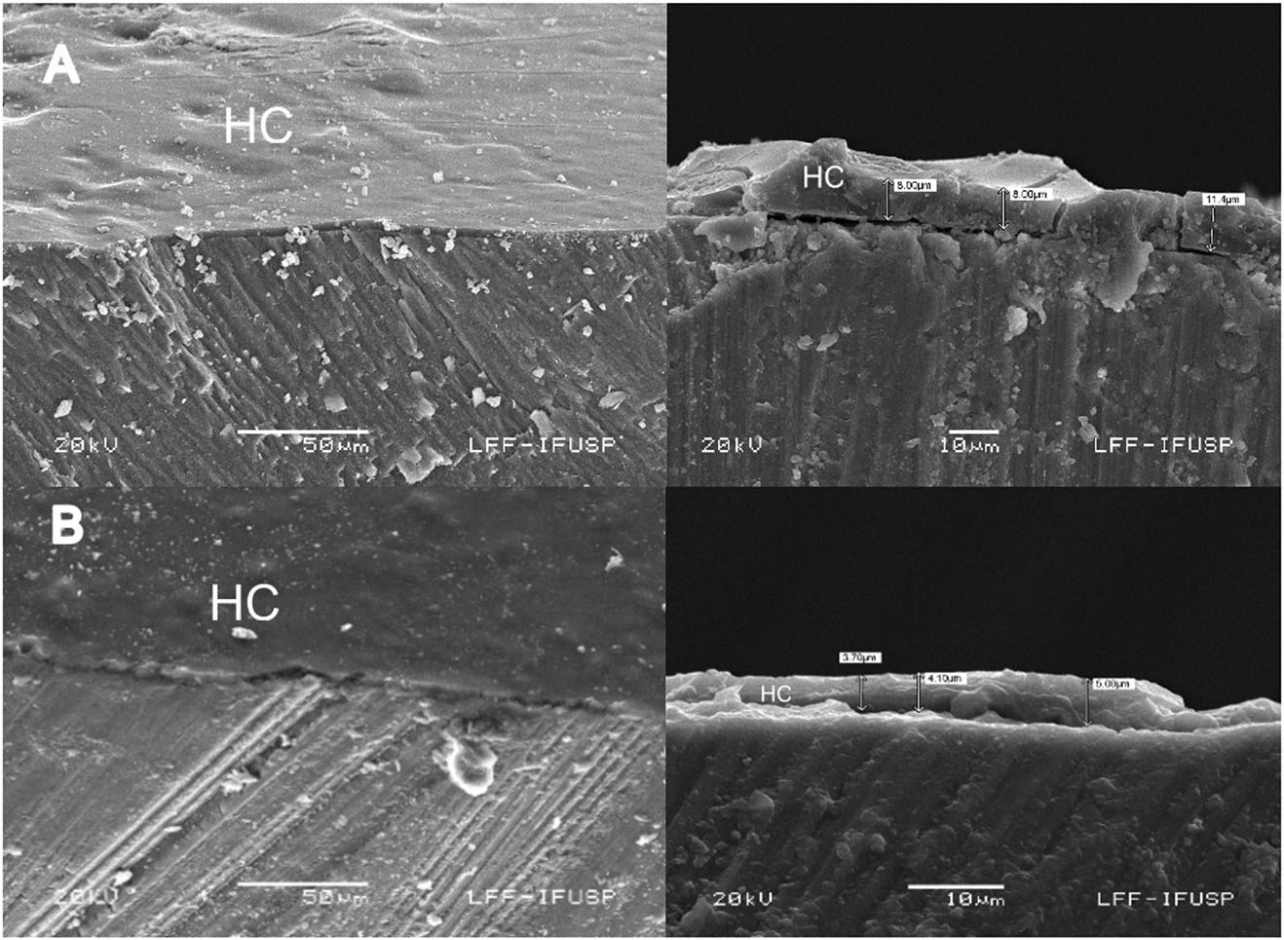
Representative cross-sectional SEM images of the enamel (**A**) and dentin (**B**) specimens at 500× (left images) and 2000× (right images) magnification. The thickness of the film was measured at three different randomly chosen regions. HC Hybrid Coating

In the SEM images (Fig. 3), it was possible to see presence of the hybrid coating on the enamel and dentin, shown as a thin film, with ~11 µm.

## Discussion

The first study hypothesis stated that the hybrid coating would protect the dental substrates against dental erosion and erosion–abrasion. It was partially accepted, because the hybrid coating was able to reduce SL under dental erosion, but not under erosion–abrasion. The microscope images showed a homogenous coating covering the dental surfaces. The cracks visualized in the images may have resulted from the procedures of specimen preparation for SEM. In a preliminary test (unpublished data), the profilometry analysis showed that the varnish was able to form a layer on dental surfaces, the mean thickness of this layer on enamel was of 2.3 µm (±2.1); while on dentin, it was of 2.6 µm (±1.7). The release test showed that fluoride and stannous were able be encapsulated and the clays released the content on both media. On stannous release analysis, in saliva, after 1-h release, no further significant increase occurred for the Sn^2+^ concentration; on the contrary, the cumulative concentration decreased. This could be related to the cations present in the artificial saliva formulation, such as Na^+^. As the Sn^2+^ release occurs due to ionic exchange, the higher cation concentration on the medium allowed part of the Sn^2+^ to return to the bentonite.

In the erosion model on enamel, the hybrid coating without additives (HC) was able to promote significant protection against dental erosion, similar to that achieved by the positive control (Fluor Protector). This result suggested that the hybrid coating was able to act as a mechanical barrier against the action of the erosive acids on this substrate. However, the fact that some specimens of the HC group showed a discreet SL on conclusion of the cycling protocol, implied that the hybrid coating may have been partially removed from some areas during the course of cycling. For dentin, the hybrid coating was also effective, matching the protection offered by the positive control.

Hybrid coatings are usually prepared by the sol–gel process, which is a low cost and simple method for obtaining coatings, and it is based on the hydrolysis and condensation reaction of metal alkoxides, such as tetraethoxysilane TEOS. Hybrid coatings are prepared from the sol of alkoxide mixed with organic polymers or organoalkoxysilanes, such as GPTMS (3-glicidoxypropyltrimethoxy silane) and γ-APS (gamma-aminopropyltrimethoxysilane) [13], which derive their organic characteristics from the epoxy and amine groups, respectively. These groups can improve the crosslinking ability of the hybrid coatings, and their mechanical properties [13]. In addition, the incorporation of organic components into inorganic materials allows a wide range of possibilities of functionalization and tailoring, such as for example, targeting an improvement in their optical properties [26].

The ability of the hybrid coatings to prevent metal corrosion is known to be strongly dependent on their thickness and structural quality [13, 27]. The material will achieve good properties with an adequate cure, which should be performed at high temperatures [28] that will evaporate the solvent of the hybrid coatings, promote continued polycondensation, decomposition of organics matter by oxidation, and the collapse of the pores [13]. However, since it is unfeasible to use high temperatures under in vivo conditions, we conducted preliminary tests to check how the hybrid coating could be cured with the use of items of equipment that are commonly available in dental offices. We decided to use the heat from a polywave LED light curing unit with irradiance of 1000 mW/cm^2^. This was shown to be capable of curing the hybrid solution in a satisfactory manner, because after irradiation, a dry layer of the hybrid coating was observed on the tooth surfaces. Nevertheless, it could be suggested that the anti-erosive properties of the hybrid coating could still be enhanced, for a more prolonged effect, by making improvements in its cure.

Another aspect that should be considered was the application of the hybrid coating on previously eroded specimens. This was performed because this treatment would most likely be suggested for patients that already presented signs of erosive tooth wear. However, it could be hypothesized that the initial lesion created was too severe, leaving a fragile and easy-to-remove remaining layer of tissue, and this could have interfered in the hybrid coating adhesion to enamel. It could also be suggested that the hybrid coating adhered to the eroded enamel; however, because of the enamel fragile state, they were both removed during the subsequent cycles.

One alternative to be tested in future studies would be to promote a surface etching treatment on the eroded enamel—similar to the procedure performed for the adhesion of composites—prior to the hybrid application. This could potentially induce a more controlled micro retention of the hybrid coating on the surface, improving its adhesion.

A previous investigation by our group showed that the hybrid coating (from a diluted hybrid solution similar to the type used here) could flow into the more porous and irregular structure of dentin, penetrating into its tubules, causing a reduction in dentin permeability [29]. In this previous study, the hybrid coating remained on the dentin surface even after using erosive and abrasive models conducted separately. The advantages of occluding the dentinal tubules with the hybrid coatings would be that this material is thin and transparent, with less possibility of over contouring when applied in the cervical region of the tooth. This would be of relevance especially in the cases of subjects with dentin hypersensitivity associated with gingival recession and minor tooth substance loss.

Nonetheless, for the two substrates tested, similar to the condition observed for the positive control, the hybrid coating was not able to resist the combination of erosive and abrasive models. In addition to searching for methods to improve the cure of the hybrid solution in the oral cavity (one of which could be related to the addition of photo initiators of polymerization in its composition, thereby helping to obtain a better cure of the hybrid coating), considering the mechanical forces that are constantly acting in this environment, an improvement in the mechanical properties of the hybrid coating is also required. One suggestion would be addition of fillers, as it is known that they can influence the mechanical properties of the material [30]. It should be taken into account, however, that all these modifications can change other aspects of the hybrid coatings, such as film thickness, its color, and viscosity. Thus, further investigations are needed to evaluate the impact of these changes on the properties of the material.

The second hypothesis of our study, to test whether the hybrid coatings modified with fluoride and stannous would show improved protection against dental erosion and erosion–abrasion when compared with the non-modified hybrid coating was rejected. Unexpectedly, for the two substrates, the hybrid coatings loaded with fluoride and fluoride plus stannous, either encapsulated or not, did not show any higher ability to prevent tooth erosion or erosion–abrasion than the non-modified hybrid coating.

Although the hybrid coatings were observed to form a homogenous film on the dental surfaces, corrosive ions are known to have the potential capacity for diffusing through the coating pores [31], eroding the dental substrates underneath. Porosities will always be present in the hybrid coatings, but the addition of agents that inhibit corrosion can protect the areas in which the integrity of the coating was compromised [28]. These agents need to be released slowly, for the long term protection of the defects [32]. For this purpose, encapsulation of these agents is important, because this can protect the agent, avoiding its interaction with the sol–gel matrix. These agents would then be released, for example, in response to changes in pH of the environment [28]. Halloysite is a negatively charged aluminosilicate, with a tubular structure with an average diameter of 50 nm and length of 500–700 nm, which can store corrosion inhibitors and release them by a stimulus of their own corrosion process [33]. This was the material of choice to store the F^−^ in the present study. Bentonite is an absorbent aluminum phyllosilicate clay consisting mostly of montmorillonite, which can be found in the shape of plaques or laminas. When in plaques, stacking of the layers leaves an interlayer space (or galleries) between them, with exchangeable cations, such as Na^+^, Ca^2+^, Li^+^ [34]. Due to its high cation exchange capacity, resulting from isomorphic substitution, bentonite was chosen as the material to encapsulate Sn^2+^.

While the addition of fillers can improve the mechanical resistance of coatings, if in excess, they may jeopardize its action as a physical barrier, in addition to making the films more opaque [28, 35]. We suggest that this occurred in the present study, as the modified films were more viscous after the addition of the encapsulated material. This could have compromised their properties, making it easier for the films to detach from the dental surfaces, thereby leaving little room for the encapsulated ions to act.

As far as the non-encapsulated fluoride and stannous effect was concerned, it could be suggested that because the hybrid coating was a dense network, it did not allow for the release of these agents in sufficient amounts to significantly prevent dental erosion. In addition, the dental erosion cycling model adopted was relatively aggressive, with several episodes of acid challenges, simulating clinical conditions of high risk for erosive tooth wear. This could have further contributed to the lack of effect of fluoride and stannous.

The varnish Fluor Protector, used as the positive control, only showed a protective effect against dental erosion and not against erosion–abrasion. This material has difluorsilane and polyurethane in its composition, the latter being the component responsible for allowing the intimate contact with tooth surfaces, thereby promoting more efficient adhesion to the substrate than that of a resin-based varnish [36–38]. A previous investigation on dentin substrate showed that Fluor Protector was intact after tooth erosion, but partially detached after tooth erosion–abrasion [38], suggesting that it had lower resistance to the mechanical action of toothbrushing.

## Conclusions

Considering the limitations of this in vitro investigation, it could be concluded that the hybrid coating was able to protect the tooth surfaces against dental erosion, with upward trend for a better effect on dentin. However, further studies are needed to enhance its mechanical properties, so that it can effectively resist both erosive and abrasive models. The addition of anti-erosive compounds did not enhance the protective effect of the hybrid coating.
